# Increase in the Immune Response in Balb/c Mice after the Co-Administration of a Vector-Based COVID-19 Vaccine with Cytosine Phosphoguanine Oligodeoxynucleotide

**DOI:** 10.3390/vaccines11010053

**Published:** 2022-12-26

**Authors:** Divine Ainee Celise, James Kimotho, Josephine W. Kimani, Alex Kigundu Muriithi, Eddy Okoth Odari

**Affiliations:** 1 Department of Molecular Biology and Biotechnology, Pan African University Institute for Basic Sciences, Technology and Innovation (PAUSTI), P.O. Box 62000-00200, Nairobi, Kenya; 2 Innovation and Technology Transfer Division, Kenya Medical Research Institute (KEMRI), P.O Box 54840-00200, Nairobi, Kenya; 3 School of Biomedical Sciences, Jomo Kenyatta University of Agriculture and Technology (JKUAT), P.O. Box 62000-00200, Nairobi, Kenya; 4 School of Pharmacy, Jomo Kenyatta University of Agriculture and Technology (JKUAT), P.O. Box 62000-00200, Nairobi, Kenya

**Keywords:** severe acute respiratory syndrome coronavirus 2 (SARS-CoV-2), viral vector-based vaccine, adjuvants, CpG ODNs

## Abstract

The effects of cytosine phosphoguanine oligodeoxynucleotides (CPG ODNs) on immune response have been demonstrated for different vaccines; however, such information is limited for the vector-based Coronavirus disease 2019 (COVID-19). This paper aims to demonstrate the potential effect of CPG ODNs on immunological response against the vector-based COVID-19 vaccine on Balb/c mice using a JNJ-78436735 Ad26.COV2-S recombinant as a model vaccine. A total of 18 BALB/c mice clustered into six groups were used. All groups were observed for 14- and 28-days post immunization. Qualitative determination of IgG was performed using indirect Enzyme-Linked Immunosorbent Assay (ELISA) and qPCR for cytokine profiling. A significant (*p* ≤ 0.001) rise in antibody response was observed for groups 3 and 4, who also showed increased expression levels of Tumor Necrosis Factor (TNF) and Interferon Gamma (IFN-γ). Immunological parameters for toxicity were normal in all treatment groups. We conclude that supplementing vector-based COVID-19 vaccines with CpG ODNs has the potential to boost the body’s immune responses to severe acute respiratory syndrome coronavirus 2 (SARS-CoV-2) infection.

## 1. Introduction

The 2019 coronavirus disease (COVID-19) pandemic is a public health concern that has not only led to high morbidity and mortality in the populations, but has negatively affected the global economy [1,2]. The health and economic burden caused by this pandemic therefore calls for an urgent launch of effective measures against SARS- CoV-2 [2], with the aim of ending the pandemic, or reducing its intensity on the economy and disease severity in the populations [3,4]. The spike protein has been demonstrated to be the most effective SARS-CoV-2 antigen and is thought to be the crucial target for SARS-CoV-2 vaccines [5,6]. The development of vaccines against SARS-CoV-2 is a strategy for preventing and ending the pandemic where different vaccine technologies have been developed such as inactivated vaccines, recombinant protein vaccines, live-attenuated vaccines, viral vectors (adenovirus) vaccines, DNA vaccines, and mRNA vaccines [5]. Adenovirus-vector-based vaccines elicit powerful immunological responses due to the presence of viral proteins and the stimulation of an innate immune response [6].

Angiotensin-Converting Enzyme 2 (ACE2) and the Receptor-Binding Domain (RBD) of the spike glycoprotein are both present on the surface of human host cells, and their interactions with the spike receptor glycoproteins of SARS-CoV-2 allow the virus to infect and enter the cells causing infection [7]. The spike glycoprotein of SARS-CoV-2 is therefore an appropriate target for a vaccine owing to the viral mechanism of cell invasion [8]. Clinical trials have shown that recipients of a single dose of the COVID-19 Janssen Vaccine were 67% protected against symptoms of SARS-CoV-2 infection, 77% from severe/critical COVID-19 at 14 days, 85% protected after 28 days, and 93% were protected against hospitalizations [9]. Lower vaccine efficacy has been noted however with the development of new variants [10]. To boost the development of protective and therapeutic vaccination responses, it is desirable to find adjuvants that could help antigen-presenting cells (APC) to stimulate CD8+ T-cell responses in the absence of T-cell help [11].

The cytosine phosphoguanine oligodeoxynucleotides (CpG ODN) have been shown to maximize immune response [12]. They are single-stranded synthetic DNA molecules with unmethylated CpG dinucleotides in specific sequence contexts (CpG motifs) [13]. They can cause Toll-like Receptor-9 (TLR-9)-expressing cells, such as human plasmacytoid dendritic cells and B cells, to produce an innate immune response that includes the production of T helper-1 and proinflammatory cytokines [14]. Their potential as a vaccine adjuvant has been proven in trials using ovalbumin, heterologous gammaglobulin, and hen egg lysozyme as model antigens [15]. In all the latter studies it has been demonstrated that CpG ODN is a greater Th1-like adjuvant compared to the “gold standard” (complete Freund’s adjuvant (CFA)) based on its capacity to promote the development of Interferon-secreting T cells and cytotoxic T cells. Furthermore, other studies have demonstrated that CpG ODN achieved an increased antigen-specific activation level without triggering any of the severe local inflammatory effects seen with CFA [16]. Co-administering CpG ODNs with vaccines boosts the formation of humoral and cellular vaccine-specific immune responses and improves the activity of professional antigen-presenting cells [17].

In this study, an increased effect of the CpG oligodeoxynucleotides (ODNs) adjuvant on immunological response against viral vector-based SARS-CoV-2 vaccine was demonstrated in BALB/c mice.

## 2. Materials and Methods

### 2.1. Study Approval

Approval for animal use was obtained from the Mount Kenya University scientific Review Committee. (Approval number 1386).

### 2.2. Animal Model, Sample Stratification, and Immunization

A total of 18 female BALB/c mice (20 ± 2 g) purchased from the Institute of Primate Research (IPR) in Kenya were used in this study. The mice were acclimated for seven (7) days in a standard facility at the Kenya Medical Research Institute (KEMRI). During this time, mice were fed on commercial pellets and had access to water. They were clustered into 6 groups. Mice in group 1 and group 2 received a single subcutaneous injection of 4 × 10^9^ VP and 8 × 10^9^ VP Janssen vaccine (JNJ-78436735 Ad26.COV2-S, recombinant; Janssen Pharmaceutical Companies, USA), respectively. Mice in group 3 and group 4 first received a single subcutaneous injection of 4 × 10^9^ VP and 8 × 10^9^ VP Janssen vaccine, respectively, followed by a single dose of 0.1 nM/µL of CpG ODNs (Table 1). Group 5 mice received a single dose of 0.1 nM/µL of CpG ODNs while group 6 mice received 1X PBS.

### 2.3. Sample Collection

Mice were bled on day 14 and day 28 after vaccination, and the blood was separated to obtain plasma or retained as whole blood. Blood for plasma was collected by bleeding from the tail whereas whole blood was collected via cardiac puncture. The tail blood was mixed with the heparin injection in a 1.25 mL cryovial tube, centrifuged at 1400 rpm for 10 min, and the supernatant was collected. Before collection of the whole blood, mice were euthanized using CO_2_ and 700 μL collected in 1.5 mL EDTA-containing collection tubes. Plasma and whole blood collected were stored at −20 °C and −80 °C, respectively. Moreover, spleen tissues were obtained by dissecting the mice and placing the spleen in sterile 1.5 mL Eppendorf tubes while still on ice. The spleen samples were stored at −80 °C before they were used for total RNA extraction and other downstream experiments.

### 2.4. Evaluation of Humoral Immune Responses to the Vaccine

Enzyme Linked Immunosorbent Assay (ELISA) was used to determine SARS-CoV-2 anti-spike IgG levels using the Mouse Anti-SARS-CoV-2 Spike Protein Antibody IgG Titer Serologic ELISA Kit (Solarbio Science & Technology Co., Ltd., Beijing, China). The ELISA plates were read at 450 nm using the VersaMax™ ELISA Microplate Reader (Sunnyvale, CA, USA). Samples from each mouse were assayed in duplicate and the mean OD (Optical Density) value was used to represent each experimental unit.

### 2.5. mRNA Expression of TNF and INF-γ on Immunized Mice

Total RNA was extracted using the Total RNA Extraction Kit (Solarbio Science & Technology Co., Ltd., Beijing, China) as per the manufacturer’s instructions. The RNA concentration and purity were assessed using the NanoDrop™ 2000/2000c spectrophotometer (Thermo Fisher Scientific, Waltham, MA, USA) at absorbance 260/280 and samples stored at −80 °C for further experiments. Synthesis of cDNA from extracted total RNA samples was completed using the Universal RT-PCR Kit and protocol (Solarbio Science & Technology (Beijing, China).

### 2.6. Quantitative Polymerase Chain Reaction (qPCR)

A 2X SYBR Green quantitative PCR Mastermix (Solarbio Science & Technology Co., Ltd., Beijing, China) was used in a 25μL total reaction volume (Table 2). Amplification was completed on the Applied Biosystems Quant Studio 5 (PE Applied Biosystems, Waltham, MA, USA) qPCR platform (Table 2). The primers used to amplify TNF, INF-γ, and the housekeeping genes were as shown in Table 3. Relative quantification of gene expression was calculated using the delta–delta threshold cycle (∆∆Ct) formula ∆Ct = Ct (gene of interest)—Ct (housekeeping gene) according to [17].

### 2.7. Assessment of Hematological Profiles and Biochemical Tests

Whole blood count analysis was performed using the HumaCount 30^TS^ (Human Diagnostics Worldwide, Wiesbaden, Germany) hematology analyzer machine following the laboratory protocol [19]. The serum levels of Aspartate Transaminase (AST), Alanine Transferase (ALT), Gamma-Glutamyltransfearse (GGT), Creatinine, and Urea were determined using the Reflotron colorimetric test strips (Woodley Equipment Company, Lancashire, England) and protocols.

### 2.8. Data Analysis

Antibody levels of the vaccinated groups and the control groups were compared using a one-way analysis of variance (ANOVA) 1-factor test for three or more groups and *t*-tests for two (2) groups were used to determine the significant differences by using GraphPad prism software. Values were considered significant at 95% confidence intervals (*p* ≤ 0.05).

## 3. Results

### 3.1. Determination of IgG Antibodies

#### 3.1.1. Reliability Testing for the Results Obtained

For each group, a minimum of three mice were inoculated with similar concentrations and a coefficient of variation among the different doses was recorded (Table 4 and Table 5). It was noted that the use of Vac 40/CPG and Vac 80/CPG was reliable, whether on day 14 (CV = 0.6% and CV = 1.6%) or on day 28 (CV = 3.8% and CV = 2.0%), respectively (Table 4 and Table 5).

#### 3.1.2. Levels of IgG Generated within Different Experimental Groups

**Figure 1:**
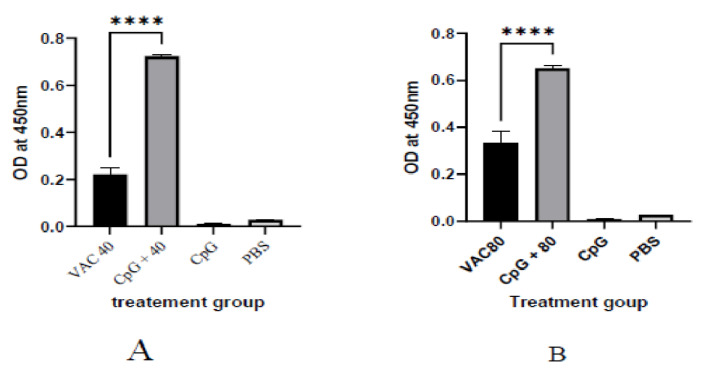
The IgG antibody immune response to SARS-CoV-2 at 14 days after immunization. (**A**) The results of SARS-CoV-2 spike protein-specific IgG antibodies in the plasma of 4 × 10^9^ VP of vaccine plus the CpG ODNS group showed statistical significance (*p* ≤ 0.001), 2-fold increase in IgG against 4 × 10^9^ VP of vaccine. (**B**) The results of SARS-CoV-2 spike protein-specific IgG antibodies in the plasma of 8 × 10^9^ VP of vaccine plus the CpG ODNs group. There was a 1.5-fold increase for the CPG-supplemented against 8 × 10^9^ VP of the vaccine-only group. A 95% CI was considered for all tests. (**** *p* < 0.0001).

**Figure 2:**
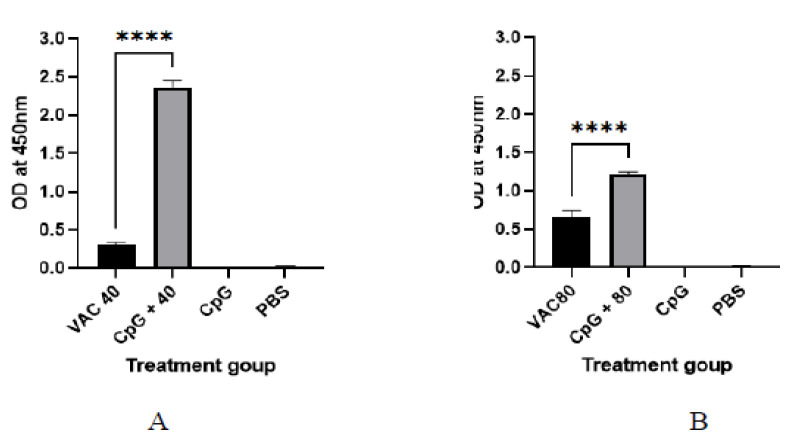
The IgG antibody immune response to SARS-CoV-2 at 28 days after immunization. (**A**) The results of SARS-CoV-2 spike protein-specific IgG antibodies in the plasma of 4 × 10^9^ VP of vaccine plus the CpG ODNS group showed statistical significance (*p* ≤ 0.001) with a 5.6-fold increase in IgG against 4 × 10^9^ VP of vaccine. (**B**) The results of SARS-CoV-2 spike protein-specific IgG antibodies in the plasma of 8 × 10^9^ VP of vaccine plus the CpG ODNs group. There was a 1.2-fold increase against 8 × 10^9^ VP of the vaccine group. A 95% CI was considered for all tests. A 95% CI was considered for all tests. (**** *p* < 0.0001).

It was shown that among the 4 × 10^9^ VP group at day 14, a statistically significant (*p* ≤ 0.001), 2-fold increase in IgG was shown for the CPG-supplemented group (OD = 0.7) against the vaccine-only group (OD = 0.22). On the other hand, only about a 1.5-fold increase for the CPG-supplemented against vaccine-only group (OD = 0.33 versus OD = 0.55) was shown among the 4 × 10^9^ VP group (Figure 1). At day 28, the group of 4 × 10^9^ VP was statistically significant (*p* ≤ 0.001) where a 5.6-fold increase in IgG was shown for the supplemented group (OD = 1.7) against the vaccine-only group (OD = 0.3). On the other hand, there was only a 1.2-fold increase for the supplemented group (OD = 0.7) against the vaccine-only group (0.6) observed among the 8 × 10^9^ VP group (Figure 2).

**Figure 3:**
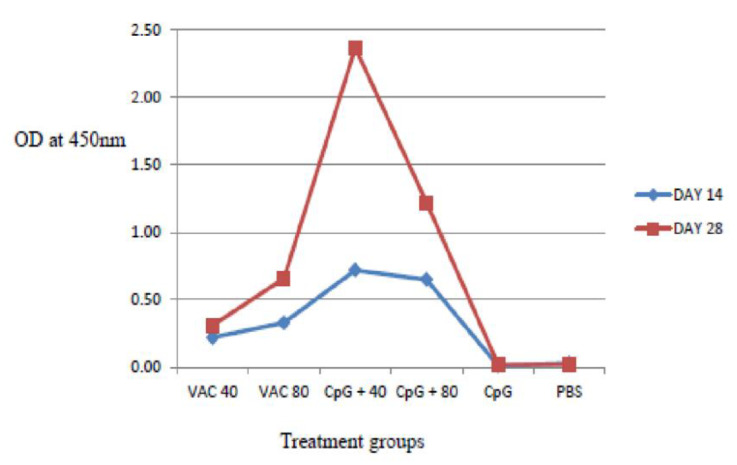
IgG antibody response to SARS-CoV-2 using ELISA assays on days 14 and 28. The IgG response was significantly stronger in the 4 × 10^9^ VP of vaccine supplemented by CpG ODNs mice, compared to the CpG ODNs and 8 × 10^9^ VP of vaccinated mice (*p* < 0.06).

IgG antibody titers on day 28 were found to be higher compared to day 14 both in the vaccine-only and the vaccine plus CpG ODNs groups. The IgG response was significantly stronger in the 4 × 10^9^ VP of vaccine supplemented by CpG ODNs mice, compared to the CpG ODNs and 8 × 10^9^ VP of vaccinated mice (*p* < 0.06) (Figure 3). For the 8 × 10^9^ VP vaccine concentration, high IgG antibody titers were shown compared to 4 × 10^9^ VP.

### 3.2. Evaluation of mRNA Expression on Immunized Mice

**Figure 4:**
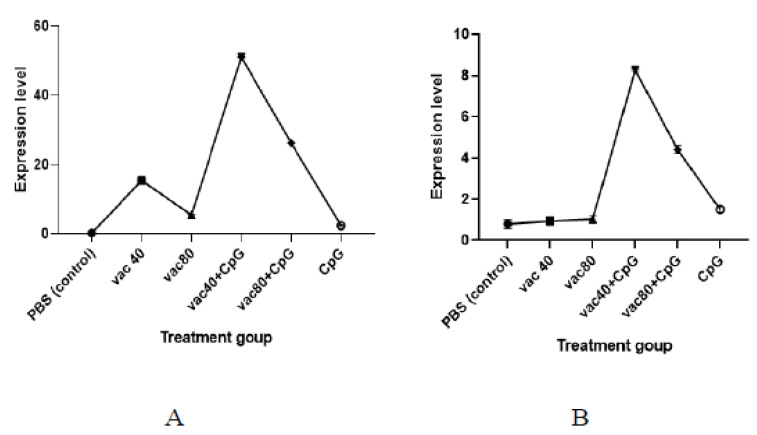
mRNA expression of TNF (**A**) and INF-γ (**B**). VACC 40 and VACC 80: groups treated with 4 × 10^9^ VP and 8 × 10^9^ VP of vaccine concentration, respectively, VACC 40 +CpG and VACC 80 + CpG: groups treated with 4 × 10^9^ VP and 8 × 10^9^ VP of vaccine plus CpG ODNs, CpG: group treated with CpG ODNs, and PBS: group treated with PBS.

A lower expression level of TNF was observed among the CpG-only treated group compared to the expression level observed for the group treated with 4 × 10^9^ VP and 8 × 10^9^ VP of vaccines (2.3 against 15.4 and 5.5, respectively) (Figure 4). Similarly, the numbers of the expression level of TNF generated in mice immunized with both groups of vaccine (4 × 10^9^ VP and 8 × 10^9^ VP) plus CpG ODNs were higher (51.0 and 26.2, respectively) compared to the vaccine-only treated group. Further, the expression level of TNF for the 4 × 10^9^ VP of vaccine concentration was significantly higher compared to the 8 × 10^9^ VP of vaccine concentration (*p*< 0.0001). For INF-γ, the group of mice treated with CpG ODNs only showed a higher expression of INF-γ compared to the mice treated with 4 × 10^9^ VP and 8 × 10^9^ VP of vaccine concentrations (1.5 against 0.9 and 1.0, respectively)). Mice treated with 4 × 10^9^ VP of vaccine plus CpG showed a higher expression level compared to the mice treated with 8 × 10^9^ VP of vaccine and CpG (8.3 against 4.4) (*p* = 0.000997). The findings demonstrate the ability of CpG ODNs to enhance the secretion of TNF and INF-γ.

### 3.3. Evaluation of Hematology and Biochemical Parameters

Hematological parameters of the female BALB/c mice immunized with 4 × 10^9^ VP and 8 × 10^9^ VP of the vaccine only (group 1 and group 2), 4 × 10^9^ VP and 8 × 10^9^ VP of vaccine with CpG ODNs (group 3 and group 4), CpG ODNs only (group 5) and PBS (group 6) are shown in Table 6. Analysis of RBC revealed that group 2 was 1.1 times higher than all other groups; all the parameters for erythrocyte were within the range of normal untreated female BALB/c mice (Table 6). Higher platelet counts were observed in vaccine of 4 × 10^9^ VP supplemented with CpG ODN (group 4) with lower counts observed in the vaccine-only group of 4 × 10^9^ VP. Leukocyte count showed that the vaccine group of 4 × 10^9^ VP supplemented with CpG ODN had a higher number of immune cells (WBC) and in all groups, no basophils were found. Overall analysis showed no statistical difference (*p* ≥ 0.99) between treated mice and untreated mice.

**Figure 5:**
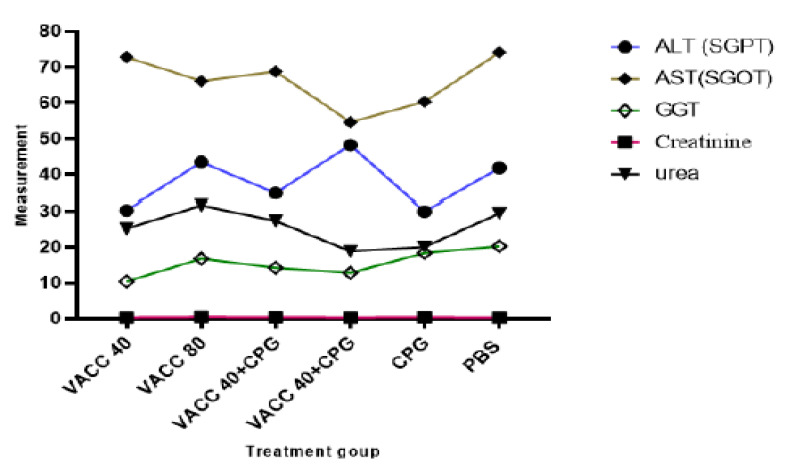
Results of biochemical analysis. VACC 40 and VACC 80: groups treated with 4 × 10^9^ VP and 8 × 10^9^ VP of vaccine concentration, respectively, VACC 40 +CpG and VACC 80 + CpG: groups treated with 4 × 10^9^ VP and 8 × 10^9^ VP of vaccine plus CpG ODNs, CpG: group treated with CpG ODNs and PBS: group treated with PBS.

Biochemical analyses of female BALB/c mice immunized by 4 × 109 VP and 8 × 109 VP of vaccine concentration only (group 1 and group 2), 4 × 109 VP and 8 × 109 VP of vaccine with CpG ODNs (group 3 and group 4), CpG ODNs (group 5), and PBS (group 6) are presented in Figure 5. There was no statistically significant difference in biochemical parameters (ALT, AST and GGT, Creatinine, and urea) between the CPG ODNs-treated and non-treated groups. 

## 4. Discussion

The findings of this study demonstrate the potential improved efficacy of a viral vector-based vaccine against SARS-CoV-2 upon supplementation with CpG ODNs. To the best of our knowledge, this is one of the first studies to evaluate the potential of CPG ODNs to improve viral vector-based vaccines for a SARS-CoV-2 immune response. Other studies have demonstrated the use of CPG-adjuvanted anthrax vaccine-adsorbed (AVA) [20] hepatitis B vaccine [21]. In all such studies, CpG ODN demonstrated an ability to stimulate plasmacytoid dendritic cells (pDCs) and B cells which are capable of activating both innate and adaptive immune responses [22]. For instance, CpG ODN combined with AVA increased the serum IgG anti-*Bacillus anthracis* (BA) response, resulting in serum anti-BA titers that were 10-fold higher and significantly more protective by day 10 (*p*≤ 0.05) [20]. Other studies have also demonstrated that CpG ODNs have the potential to produce a Th1-biased immunological environment and enhance CD8+ T-cell responses, making them potential adjuvants for cancer vaccines [23]. 

In this study, we have demonstrated that both low-dose and high-dose JNJ-78436735 Ad26.COV2-S recombinants plus CpG ODNs produced high levels of specific IgG antibodies in comparison to vaccines only, and the revealed long-term stability of high titers of neutralizing IgG antibodies indicated that the CPG ODNs-supplemented vaccine has potential for long-lasting immune capabilities. The reliability of the CPG ODNs-supplemented vaccines was demonstrated for both concentrations of the viral particles (VP) at both 40µL and the 80µL, hence the potential stability in efficacy. The anti-SARS-CoV-2 IgG antibody is a sign of COVID-19 infection or immunization and aids in the monitoring and management of COVID-19 transmission. Neutralizing antibody titers are thus an essential evaluation index that cannot be replaced when assessing the efficiency of vaccines [24].

TNF is an important factor in the inflammatory response. This cytokine initiates the activation of several other cytokines and growth factors, as well as the recruitment of particular immune cells. Compared to other proinflammatory cytokines, TNF has been shown to be released more quickly [25]. The IFN response is triggered when host cell PRRs recognize viral PAMPs. The IFN-I response both directly and indirectly reduces viral replication [26]. Studies have demonstrated that IFN is the main agent inducing macrophage activation in lymphoid cells, an essential effector part of the adaptive immune response [27]. Multiple innate immune effector pathways, such as the release of cytokines that are often pro-inflammatory such as TNF and IL-12, and improved antigen presentation are all activated by IFN [28]. The IFN-γ is essential for controlling the adaptive immune response [29]. It is made by a wide range of lymphocytes, including regulatory T (Treg), CD4+, CD8+, B-cells, and NK cells. It strongly influences the immune system’s ability to fight off viruses and bacteria by promoting macrophage activation and antigen presentation [30]. In this study we have reported that the formulation JNJ-78436735 Ad26.COV2-S, recombinant vaccine with CpG-ODN has an improved ability to induce cellular immunity compared to the vaccine only (*p* < 0.001), as demonstrated by the higher titers of both TNF and IFN-γ for the vaccine supplemented with CPG ODNs. Both innate and adaptive immunity depend on TNF and IFN-γ, which also serve as major activators of macrophages and stimulate neutrophils and natural killer cells [29].

One main concern for DNA adjuvants, as with all adjuvants, is the problem for toxicity. Regarding ODN adjuvants, mice repeatedly treated with high doses of CpG ODN experience splenomegaly that is dose-dependent as well as additional damage from excessive immunological stimulation, including mortality [31]. Additional studies [19] have shown no apparent systemic toxicity or change in feeding, physical activity, or behavior. The findings of the hematological evaluation for all groups did not differ (*p* ≤ 0.05) for any of the parameters examined, indicating that the CpG ODNs did not affect the hematological parameters in BALB/c mice. Additionally, all groups’ values for RBC, Hematocrit, Hemoglobin, MCV, and CHCM have been shown to fall within the normal range as already published [19] for female Balb/c mice. With regard to the leukocyte differential count, the monocytes, eosinophils, and basophils had the lowest values in all groups (Table 6), showing no difference (*p* ≤ 0.05) between the groups compared to the untreated female Balb/c mice, despite their typically low levels.

Blood biochemical profiles serve as a highly valuable diagnostic tool by reflecting the physiological state of the animal [32]. Therefore, the renal biochemical profile was assessed based on the measures of urea and creatinine in order to determine if the surgical procedure affected the function of the transplant recipient organ. Additionally, the biochemical profile was assessed based on the results of liver enzymes including ALT, AST, and GGT to the animals’ overall metabolic health. The biochemical profile must be evaluated and interpreted with greater accuracy because the enzymatic profile is one of the blood parameters with the greatest variability [33]. For each group, there was no statistically significant difference compared to untreated female balb/c mice (*p* ≤ 0.05). Moreover, the amount of ALT enzyme is thought to be a preferable signal for determining the integrity of liver cells compared to AST [34], most likely due to its predominance in this organ, specifically in hepatocyte mitochondria, and longer half-life [35]. Additionally, ALT is regarded as a sign of general health in addition to being a marker of liver disorders [36]. It is thought that considerable elevations in AST levels alone are not typically indicative of liver damage [35].

## 5. Conclusions

Our study found that the co-administration of the CPG ODNs adjuvant and the Johnson and Johnson vaccine in Balb/C mice has the potential to boost humoral and cellular immune responses. The findings could contribute to the search for innovative approaches to improve the efficacy of viral vector-based COVID-19 vaccines amidst challenges such as mutations. However, further work has to be completed on the subject to be able to make scientific conclusions. Some limitations of this study include a small sample size. Even though the study produced intriguing results, the number of observations that could be made for distinct parameters was constrained by using only three female BALB/c mice per experimental unit. There is a need to conduct the study again with a larger sample size because the sample size is crucial in drawing scientific findings. Furthermore, quantitative ELISA experiments, more protective cytokines, and neutralizing antibodies could be explored in further studies.

## Figures and Tables

**Table 1:** Types of cytosine phosphoguanine oligodeoxynucleotides (CpG ODNs) used in the study.

CpG ODNs	Sequences
1828 9 (22 mer)	5′-T*CCATGACGTTC*TACTGACGT*T- 3′
18281-1 (22 mer)	5′-T*C*C*A*T*G*A*C*G*T*T*C*T*A*C*T*G*A*C* G*T*T- 3′
18281-2 (23 mer)	5′-T*GA*CTGT*GAACGTTCGGATGAT*T-3′

* Modified phosphorothioate.

**Table 2:** Quantitative Real-Time PCR Thermal Profile.

Step	Time	Temperature	Cycles
Pre-Denaturation	10 Minutes	95 °C	1
Denaturation	15 Seconds	95 °C	45
Annealing/Extension	1 Minute	60 °C	

**Table 3:** Primers and expected Amplicon sizes for TNF and INF-γ toxicity analysis.

Cytokine	Forward Primer 5′--3′	Reverse Primer 5′--3′	NCBI	Size of Amplicon	Reference
Tumor necrosis factor (TNF)	CTCCAGGCGGTGCCTATGT	GAAGAGCGTGGTGGCCC	NM_013693.3	76 bp	[18]
Interferon Gamma (IFN-γ)	CTGATTTCAACTTCTTTGGCT	TATCCGCTACATCTGAATGA	NM_008337.4	136 bp	[18]
*HPRT1* (Hypoxanthine guanine phosphoribosyl transferase 1)	TCCTCCTCAGACCGCTTTT	CCTGGTTCATCATCGCTAATC	NM_013556.2	90 bp	[17]

**Table 4:** Reliability of the vaccine concentrations among BALB/c mice at day 14.

Compound/Molecule Dose	Optical Density (OD) Signals Generates			Coefficient of Variation %
	Mouse 1	Mouse 2	Mouse 3	
Vac 40	0.239	0.194	0.234	11.1
Vac 80	0.383	0.329	0.291	13.8
Vac 40/CPG	0.721	0.723	0.729	0.6
Vac 80/CPG	0.643	0.663	0.647	1.6
CPG	0.01	0.01	0.01	15.7
PBS	0.03	0.03	0.03	3.4

Abbreviations: VACC 40 and VACC 80: groups treated with 4 × 10^9^ VP and 8 × 10^9^ VP of vaccine concentration, respectively, VACC 40 +CpG and VACC 80 + CpG: groups treated with 4 × 10^9^ VP and 8 × 10^9^ VP of vaccine plus CpG ODNs, CpG: group treated with CpG ODNs, and PBS: group treated with PBS.

**Table 5:** Reliability of the vaccine concentrations among BALB/c mice at day 28.

Compound/Molecule Dose	Optical Density (OD) Signals Generates			Coefficient of Variation %
	Mouse 1	Mouse 2	Mouse 3	
Vac 40	0.329	0.323	0.272	10.2
Vac 80	0.6835	0.724	0.579	11.3
Vac 40/CPG	2.3095	2.472	2.322	3.8
Vac 80/CPG	1.251	1.205	1.2145	2.0
CPG	0.0175	0.0145	0.015	10.3
PBS	0.025	0.027	0.0205	13.8

Abbreviations: VACC 40 and VACC 80: groups treated with 4 × 10^9^ VP and 8 × 10^9^ VP of vaccine concentration, respectively, VACC 40 +CpG and VACC 80 + CpG: groups treated with 4 × 10^9^ VP and 8 × 10^9^ VP of vaccine plus CpG ODNs, CpG: group treated with CpG ODNs, and PBS: group treated with PBS.

**Table 6:** Results from Hematological Profiling.

Parameters	Unity	VACC 40	VACC 80	VACC 40 + CPG	VACC 80 + CPG	CPG	PBS **
LEUKOGRAM							
WBC	*10^3^/ul	3.6	9.3	9.7	6.1	4.9	5.7
NEUTROPHILS	%	15.7	9.0	10.3	10.7	6.0	9.0
LYMPHOCYTES	%	73.7	84.7	84.0	84.3	86.7	85.7
MONOCYTES	%	9.3	5.0	4.7	5.7	6.0	6.3
EOSINOPHILS	%	1.3	1.3	1.0	1.3	1.3	1.7
BASOPHILS	%	0.0	0.0	0.0	0.0	0.0	0.0
ERYTHROGRAM							
RBC	*10^6/Ul^	8.4	9.0	8.7	8.9	7.9	7.8
HB	g/dl	16.1	15.8	15.8	15.5	13.7	14.7
HCT	%	40.2	43.2	42.5	41.8	37.1	37.3
MCV	Fl	47.9	42.7	48.9	46.6	36.7	48.1
MCH	Pg	19.1	17.6	18.1	17.3	17.3	19.0
MCHC	g/dl	39.9	36.8	37.1	37.1	36.9	39.3
PLATELETS COUNT							
PLATELETS	10^3^/ul	130.7	1003.0	1140.7	1172.0	896.0	895.7

** Standard for comparison of the range values, VACC 40 and VACC 80: groups treated with 4 × 10^9^ VP and 8 × 10^9^ VP of vaccine concentration, respectively, VACC 40 + CpG and VACC 80 + CpG: groups treated with 4 × 10^9^ VP and 8 × 10^9^ VP of vaccine plus CpG ODNs, CpG: group treated with CpG ODNs.

## Data Availability

Not applicable.
